# Relationship between outcome scores and knee laxity following total knee arthroplasty: a systematic review

**DOI:** 10.1080/17453674.2018.1554400

**Published:** 2018-12-20

**Authors:** Andreas Kappel, Mogens Laursen, Poul T Nielsen, Anders Odgaard

**Affiliations:** a Department of Orthopedic Surgery/Clinical Institute, Aalborg University Hospital, Aalborg, Denmark;; b Department of Orthopedic Surgery, Copenhagen University Hospital Herlev-Gentofte, Hellerup, Denmark

## Abstract

Background and purpose — Instability following primary total knee arthroplasty (TKA) is, according to all national registries, one of the major failure mechanisms leading to revision surgery. However, the range of soft-tissue laxity that favors both pain relief and optimal knee function following TKA remains unclear. We reviewed current evidence on the relationship between instrumented knee laxity measured postoperatively and outcome scores following primary TKA.

Patients and methods — We conducted a systematic search of PubMed, Embase, and Cochrane databases to identify relevant studies, which were cross-referenced using Web of Science.

Results — 14 eligible studies were identified; all were methodologically similar. Both sagittal and coronal laxity measurement were reported; 6 studies reported on measurement in both extension and flexion. In knee extension from 0° to 30° none of 11 studies could establish statistically significant association between laxity and outcome scores. In flexion from 60° to 90° 6 of 9 studies found statistically significant association. Favorable results were reported for posterior cruciate retaining (CR) knees with sagittal laxity between 5 and 10 mm at 75–80° and for knees with medial coronal laxity below 4° in 80–90° of flexion.

Interpretation — In order to improve outcome following TKA careful measuring and adjusting of ligament laxity intraoperatively seems important. Future studies using newer outcome scores supplemented by performance-based scores may complement current evidence.

Modifiable surgical factors influence the outcome of TKA procedures. Implant alignment, soft tissue balancing, and choice of implant constraint is dependent on preoperative anatomical conditions, surgical technique, and the experience, preference, and thoroughness of the surgeon. Implant alignment is known to affect both revision rate (Ritter et al. 2011, Gromov et al. 2014, Kim et al. 2014, Lee et al. 2018) and outcome (Longstaff et al. 2009, Huang et al. 2012, Gromov et al. 2014), but the influence of soft tissue laxity is not as well described and has not previously been the subject of systematic review.

In TKA surgery, knee laxity is evaluated both intraoperatively and at follow-up. Different surgical techniques to obtain optimal soft tissue balance have been described (Babazadeh et al. 2009, Mihalko et al. 2009), and numerous tools, such as computer-assisted surgery, trial insert sensors, tensioners, spreaders, spatulas and spacer blocks, have been developed to assist the surgeon quantify intraoperative laxity. However, intraoperative evaluation of soft tissue laxity is still challenging and can among other factors be influenced by the position of the patella, muscular tension, and the external load on the knee. Furthermore, the laxity measured with trial implants might change following implantation of the final implant (Nodzo et al. 2017) and the ligament tension might change following surgery. In most cases soft-tissue balance is based on subjective assessment, and therefore depends on the individual surgeon’s experience and preferences.

Clinical evaluation of knee laxity at follow-up has low levels of intra- and inter-observer reliability (Liow et al. 2000), and the clinical evaluation might also be biased by patient complaints. Methods for instrumented laxity measurements are available but not incorporated in clinical practice on a large scale, and a gold standard on instrumented laxity measurement following TKA surgery has not yet been established. The range of soft-tissue laxity that favors both pain relief and optimal knee function following TKA remains unclear.

The objective of this systematic review is to clarify evidence regarding the relationship between objectively quantifiable soft tissue laxity at follow-up and outcome scores in primary TKA.

## Methods

We searched the PubMed, Embase, and Cochrane databases for combinations of search words describing knee arthroplasty, soft tissue laxity, and outcome to identify papers reporting on the relationship between knee laxity and outcome following primary TKA. Only studies reporting association of instrumented laxity measurement following primary TKA and outcome scores were included. Studies comparing laxity of specific implants were included only if laxity measurements were analyzed with respect to outcome scores. Studies reporting manual, non-instrumented laxity measurements were not included. Range of motion (ROM) was not considered an outcome score. For the full search history, see Supplementary data. The search was performed in June 2017 and updated in January 2018. Additional papers were added based on references; for all the included studies cross-references were identified and reviewed using Web of Science. AK conducted the primary review of the search results and the identification of full-text articles for assessment. All authors participated in the assessment of full-text articles and a minimum of 2 authors would agree on inclusion of studies in the analysis; disagreement was solved by consensus. The corresponding author independently completed data extraction according to the study protocol and data were verified by a second reviewer; disagreement was solved by consensus.

Quality of the included studies was assessed using the Methodological Index for Non Randomized Studies (MINORS).

### Registration, funding, and potential conflicts of interest

The Preferred Reporting Items for Systematic Reviews and Meta-Analyses (PRISMA) statement was followed when performing this review. The review protocol was published on the PROSPERO database in July 2017, with registration number CRD42017069779 (https://www.crd.york.ac.uk/PROSPERO/). The protocol was updated in April 2018. The authors’ institutions funded the study. No conflict of interest declared.

## Results

After removal of duplicates 3,228 studies were screened based on title, abstract, and in some cases full text. 34 full-text articles were assessed for eligibility and 14 articles fulfilled the criteria (Yamakado et al. 2003, Kuster et al. 2004, Ishii et al. 2005, Jones et al. 2006, Seon et al. 2007, Van Hal et al. 2007, Seon et al. 2010, Schuster et al. 2011, Seah et al. 2012, Nakahara et al. 2015, Oh et al. 2015, Graff et al. 2016, Tsukiyama et al. 2017, Matsumoto et al. 2017) (Figure).

**Figure:**
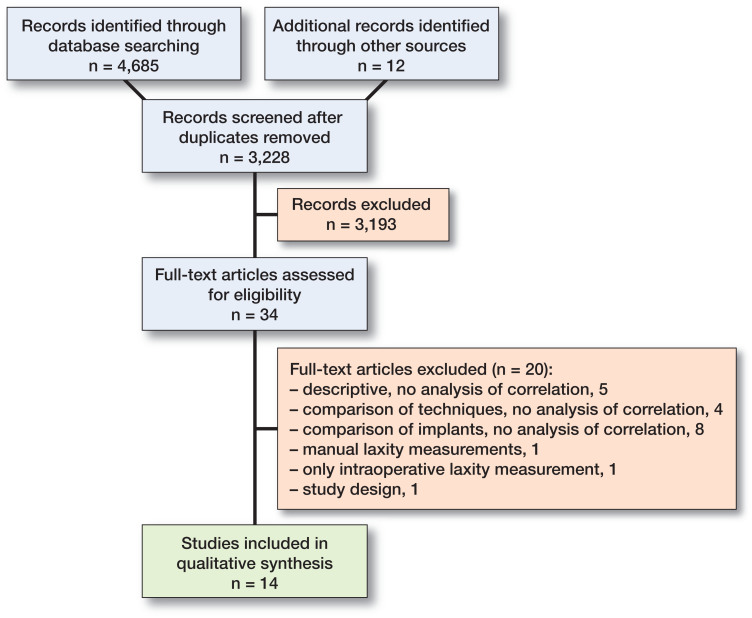
Flowchart demonstrating the PRISMA technique used to evaluate the studies

All eligible studies were cohort studies and methodologically quite similar with follow-up examination on a cohort of uncomplicated and non-revised primary total knee arthroplasties with measurement of laxity and outcome. Level of evidence according to “The Oxford 2011 Levels of Evidence” (http://www.cebm.net/index.aspx?o=5653) was level III or below. The studies obtained MINORS scores from 9 to 13; the maximum score is 16. Most commonly low scores were obtained for criteriona 2 (Inclusion of consecutive patients), criterion 5 (Unbiased assessment) and criterion 8 (Prospective calculation of the study size) (Table 1, see Supplementary data).

The number of patients/knees ranged from 15/21 to 112/127. Mean time from surgery to follow-up was 1 year to 7 years and the mean age of the patients was 68 years to 76 years. Sex distribution varied from 43% to 95% women. Regarding surgical technique, gap-balancing dominated but 5 studies did not report details regarding technique. Intended mechanical alignment was specified in only 3 studies, all aiming for neutral mechanical alignment. All combinations of posterior cruciate retaining, posterior cruciate substituting, fixed bearing, and mobile bearing were used in the studies (Table 2).

**Table 2.** Baseline characteristics of the included studies

Author	Number of knees/patient	Follow-upyears years (range)	Mean age years (range)	Women %	Surgical technique **^a^**	Navigation %	Constrained articulation ^b^ (%)
Matsumoto	110/81	4.4 (1.1–11.5)	76 (26–91)	85	GB	18	PS-FB = 23, PS-MB = 77
Tsukiyama	50/41	4.8 (2.0–13.8)	73 (59–82)	76	–	–	PS-FB = 100
Graff	24/24	2.3 (1.0–4.8)	69 (54–80)	46	–	42	CR-FB = 100
Nakahara	94/68	4.6 (1.1–11.0)	73 (50–86)	85	MR	26	PS-FB = 100
Oh	61/61	2.2 (1.0–5.0)	68 (59–82)	79	GB	0	CR-FB = 100
Seah	100/100	2 (–)	67 (50–83)	68	GB	0	CR-FB = 100
Schuster	127/112	3.9 (0.8–5.0)	71 (50–89)	71	GB	0	CR-FB = 75, CR-MB = 25
Seon	55/55	2.8 (2.0–4.3)	68 (55–81)	84	GB	100	CR-MB = 100
Seon	42/42	1 (–)	–	95	GB	100	CR-MB = 100
van Hal	51/49	4.6 (4.1– 5.4)	73 (59–87)	76	GB	0	CR-FB = 100
Jones	97/88	7 (5.4–9.9)	70 (–)	43	–	0	CR-FB = 100
Ishii	77/71	6.4 (5.2–9.4)	77 (–)	86	–	0	CR = 69, PS = 31
Kuster	44/22	4.5 (2–7)	69 (32–82)	55	–	0	FB = 16, MB = 84
Yamakado	21/15	7.1 (4–8)	68 (58–78)	80	–	0	CR-FB = 100

**^a^**GB = gap balancing, MR = measured resection, – = not specified

**^b^**CR = posterior cruciate retaining, PS = posterior cruciate sacrificing, FB = fixed bearing, MB = mobile bearing.

Laxity measurements were performed in both the sagittal and the coronal plane, and in angulations from full extension to 90° of flexion; 7 studies reported on more than 1 condition for the measurements. Statistically significant results were not found for the 12 measurements obtained with a flexion angle between 0° and 30°, but for the 10 measurementd performed between 60° and 90°, 6 showed significant results. None of the included studies measured laxity in the range between 30° and 60° (Table 3).

**Table 3.** Methods and results of the included studies

**Author**	Soft-tissue laxity measurement	Anatomical plane, degree of flexion and mean (SD) of measurement	Outcome scores	Statistical method to compare laxity and outcome score
• Significant results				
**Matsumoto**	KS Measure Arthrometer, mean of 3 measurements	Sagittal 30°: 4.5 (2.2) mm Sagittal 60°: 3.6 (1.9) mm Sagittal 90°: 3.0 (1.9) mm	KSS, KOOS	Spearman rank correlation
• Inverse correlation between 1 of 6 KOOS sub-scores (KOOS-pain) and laxity at 60°				
**Tsukiyama**	Stress radiographs: Telos, 150 N in extension epicondylar view, 50 N in flexion	Coronal extension: Varus stress: 4.0° (2.5°) Valgus stress: 4.0° (2.4°) Coronal 80°: Varus stress: 6.2° (4.4°) Valgus stress: 3.9° (2.6°)	2011 KS	Stratification based on laxity Wilcoxon rank-sum test Pearson correlation coefficient
• 4 of 6 2011 KS sub-scores better in knees medially tight in flexion				
**Graff**	KT-1000, 89 N, mean of 3 measurements	Sagittal 20°: 3.8 (2.0) mm	OKS, KOOS, KSS, SF12	Pearson correlation coefficient
• No correlation				
**Nakahara**	Stress radiographs: Telos, 150 N	Coronal 10°: Varus stress: 5.9° (2.7°)	New KSS	Pearson correlation coefficient
• No correlation Valgus stress: 5.0° (1.6°)				
**Oh**	Stress radiographs: epicondylar view, 50 N	Coronal 90°: Varus stress: 4.7° (2.4°) Valgus stress: 4.1° (2.1°)	KSS, WOMAC	Stratification based on laxity T-test (balanced vs. unbalanced) and Kruskal–Wallis analysis
• KSS-f and WOMAC better in balanced group. (subgroups of laxity in the				
• In the balanced group KSS and WOMAC better for grade II laxity balanced group)				
**Seah**	KT-1000, 89 N, sum of anterior and posterior stress, mean of 3 measurements	Sagittal 75°: not reported	KSS, OKS, SF-36	Stratification based on laxity One-way ANOVA
• Intermediate laxity group better OKS				
**Schuster**	Rolimeter, sum of anterior and posterior stress, mean of 3 measurements	Sagittal 25°: 4.6 (2.1) mm Sagittal 90°: 4.9 (2.2) mm	KSS, VAS Pain, VAS satisfaction	Stratification based on laxity Kruskal–Wallis analysis
• No differences between groups				
Seon^2010^	Stress radiographs: Telos, 89 N, sum of anterior and posterior stress	Sagittal 90°: 8.3 mm	HSS, WOMAC	Stratification based on laxity Mann–Whitney U-test Pearson correlation coefficient
• Stable group significantly better WOMAC function				
Seon^2007^	Stress radiographs: Telos, 150 N, sagittal difference between anterior and posterior stress	Sagittal 90°: 7.1 (4.1) mm Coronal extension: Varus stress: 4.4° (2.2°) Valgus stress: 3.5° (1.4°)	m-HSS	Pearson correlation coefficient

• No correlation				
**Van Hal**	Rolimeter	Sagittal 30°: 2.8 (1.1) mm	KSS	Spearman rank correlation
• No correlation				
**Jones**	KT1000, 89 N, sum of anterior and posterior translation, mean of 3 measurements	Sagittal 30°: 7.3 (4.0) mm Sagittal 75–80°: 4.6 (3.1) mm	WOMAC, KSS, SF12	Stratification based on laxity Duncan test
• Intermediate laxity group better KSS than the large laxity group.				
**Ishii**	KT-2000, anterior force 133N, posterior force 89N, sum of anterior and posterior stress, mean of 3 measurements	Sagittal 30°: CR: 5.8 (2.9) mm, PS: 5.3 (3.2) mm Sagittal 75°: CR: 4.8 (2.3) mm, PS: 3.4 (1.5) mm	HSS	Spearman rank correlation
• No correlation				
**Kuster**	Manual stress radiographs	Coronal 30°: Varus stress: 4.3° (1.9°) Valgus stress: 4.0° (2.1°)	m-HSS, preferred knee	Stratification based on laxity T-test and chi-square
• No significance, 11 bilateral cases with a knee in each laxity group, significantly preferred laxed knee over tight knee				
**Yamakado**	KT2000, 133N, and coronal manual stress radiographs	Sagittal 30°: 9.1 (1.1) mm Coronal extension: Varus stress: 6.2° (0.9°)	m-KSS	Pearson correlation coefficient and multiple regression
• No correlation		Valgus stress: 4.3° (0.5°)		

Statistical analysis was carried out either by calculating a correlation coefficient between laxity and outcome or by stratification upon laxity followed by group comparison. The correlation coefficient was calculated in 9 studies but found to be significant in only 1. Stratification was used in 7 studies, and significant results obtained in 5. The 2 studies that did not find statistically significant correlation obtained significant results following stratification (Table 3).

Sagittal laxity measurements were done using an arthrometer in 8 studies (KT-1000 in 3 studies, KT-2000 in 2 studies [Genourob, Laval, France], Rolimeter in 2 studies [Aircast, Summit, NJ, USA], and KS measure arthrometer in 1 study [Sigmax Medical, Tokyo, Japan]) and stress radiography with the Telos device in 2 studies [Telos Arzt- und Krankenhausbedarf GmbH, Hungen, Germany]. The method resembles the drawer test and the result was measured as a distance in mm. Sagittal laxity measurements performed in the range from 60° to 90° of flexion were found to associate with outcome in 4 of 7 studies. Statistically significant correlation was found in the study by Matsumoto et al. (2017) who found correlation between laxity at 60° and 1 KOOS sub-score, i.e., KOOS pain; no correlation to laxity at 90° was found. 4 studies analyzed the results following stratification. Seon et al. (2010) measured laxity using stress radiographs at 90° and found that stable knees with laxity below 10 mm obtained better WOMAC scores. Seah et al. (2012) and Jones et al. (2006) performed the laxity measurements under equal conditions, with KT-1000 at 75–80°, and used the same stratification of the results, and both studies reported statistically significantly better outcomes for the group with laxity in the range from 5 to 10 mm. Both studies included only CR implants. Schuster et al. (2011) performed the measurements with the Rolimeter at 90° of flexion and used different limits for stratification, but did not find any significant association.

Coronal laxity was quantified using stress radiography, where the knee is opened in the coronal plane by applying manual pressure or a specified force to the medial or lateral side of the knee and the opening angle between the femoral and tibial components was measured from the radiographs. The Telos device was used in 3 studies, manual stress in 2 studies and a spring scale was used to quantify stress in 2 studies. In the 2 studies reporting measurement in angulation from 80° to 90° significant results were obtained. Tsukiyama et al. (2017) and Oh et al. (2015) reported on stress radiography in flexion using the epicondylar view with coronal stress applied and measured by a spring scale. Following stratification, significant association was obtained in both studies. Tsukiyama et al. stratified the cases into a tight group with opening angle below or equal to 3° and a loose group with larger opening angle. The medial and lateral opening angle was analyzed separately, and statistically significantly better outcome scores were obtained for the medially tight group. Oh et al. used quite another stratification. Balanced knees with numerical difference between medial and lateral opening angle being equal to or below 3° obtained the best scores. Following further stratification of the balanced knees into grades of total laxity defined as the sum of medial and lateral opening angle, statistically significantly better scores were demonstrated for the group with total laxity from 6° to 10°. In both studies mean lateral laxity (varus stress) exceeded mean medial laxity (valgus stress) (Table 3).

The most frequently used outcome score was the Knee Society Score (KSS), used in 6 studies, followed by the Western Ontario and McMaster Universities Osteoarthritis Index (WOMAC), used in 3 studies. 13 different outcome scores were used and most papers reported the use of more than one score (Table 3).

## Discussion

This systematic review deals with outcome scores and quantified measurements of soft tissue laxity following primary TKA. Any statistically significant influence of laxity in extension to limited flexion on outcome scores could not be established in the reviewed studies, but Aunan et al. (2015) found that intraoperative quantification of coronal laxity in extension correlated to KOOS when stratifying for postoperative mechanical alignment of the limb. None of the reviewed studies considered mechanical alignment in the analysis, and this result should be the subject of further research. In 6 of 9 studies measuring laxity in flexion a significant association was found, and in the studies analyzing stratified data only one study did not find significance. Hence, a correlation between outcome score and laxity in flexion must be assumed. Convincing results were found with both sagittal arthrometer measurements and coronal stress radiography. The 2 studies reporting on coronal stress radiography in flexion (Oh et al. 2015, Tsukiyama et al. 2017), found comparable mean opening angles (Table 3). In both studies the mean lateral opening angle, the result of varus stress, exceeded the mean medial opening angle, and it may cautiously be concluded that the medial opening angle should not exceed 4°. Measurement of laxity was not performed between 30° and 60° of flexion in the studies, consequently this review does not add any clarification to the discussion regarding mid-flexion instability (Vince 2016).

The included studies are all cohort studies and none are methodologically errorless, which is reflected in the MINORS scores. 2 studies that did not find statistically significant results must be assumed to be under-powered as the number of knees analyzed is quite low compared with all other studies and compared with the studies showing significant results (see Table 1). Multiple testing, with more than 1 method for laxity measurement, more than 1 outcome score or sub-score, and more than 1 statistical test, which is used in some studies, introduces the risk of false positive results (see Table 3).

The methods used to measure sagittal laxity have been validated in the non-arthroplasty knee (Lefevre et al. 2014) and 2 reports of validation following TKA were found (Matsuda et al. 1999, Mochizuki et al. 2017). 1 method for stress radiography to assess coronal laxity measurement following TKA is validated but was not used in the studies included in the review (Stähelin et al. 2003). The methods used are to the best of our knowledge not yet validated. 1 study reported validation by double measurements of 4 patients, which in this particular study equals 8 knees and 16 radiographs; the results from the double measurements are reported to lie within 1°, but the results are not described statistically (Kuster et al. 2004). Reading of angulation from coronal stress radiography is validated (Nakahara et al. 2015, Hatayama et al. 2017).

The outcome measures used in the included studies do not differ from general studies regarding TKA surgery (Theodoulou et al. 2016), with the KSS representing the most frequently used measure. However, the outcome measures used may not reveal subtle differences between knees within the range of normal surgical variation, which differ only mildly in stability. The ideal outcome scores to reveal functional differences caused by variations in soft tissue laxity should allow discrimination between patients who only undertake activities of daily life and those who perform high-demand activities like sports. The KSS is known to have a high ceiling effect, and may not reveal such differences (Na et al. 2012, Jenny et al. 2014, Aunan et al. 2016). It could be argued that the outcome measures used in some studies have not been sufficiently validated, and many of these have been surpassed by more modern outcome measures that are solely patient-reported (Behrend et al. 2012, Dawson et al. 2014). Performance-based outcome measurements of TKA patients are known to reveal functional differences that are not reflected in the outcome scores (Witvrouw et al. 2002, Stevens-Lapsley et al. 2011, Bolink et al. 2015, Naili et al. 2017). The Osteoarthritis Research Society International (OARSI) recommends that performance-based outcome measures be included to complement PROMs in future osteoarthritis research (Dobson et al. 2013). This might be of special relevance in studies investigating the impact of laxity.

To what extent knee laxity changes following surgery is debated. Changes in coronal laxity immediately following TKA implantation, stress relaxation, is described by Bellemans et al. (2006) who with the aid of computer navigation reported increased mediolateral laxity, by on average 1 mm. Matsumoto et al. (2012) measured intraoperative coronal laxity using an off-set tensor device and found correlation to 5-year follow-up stress radiography measurement in extension; however, in flexion correlation was found only for CR knees. The course of sagittal laxity is described by Mizu-uchi et al. (2006) and Schuster et al. (2011); both evaluated sagittal laxity continuously up to 5 years following cruciate retaining TKA, and significant changes in mean laxity were not detected. Regarding the course of outcome scores following TKA, a recent review reported no different in outcome scores between 12 months’ and 24 months’ follow-up when using KSS or WOMAC (Ramkumar et al. 2018). The mean follow-up period in the studies we reviewed ranged from 1 year to 7 years and seems appropriate, but the large range that is present in some of the studies might introduce bias.

Differences in soft tissue laxity between specific implants and concepts of constraint might blur this review as conflicting results are reported regarding both the influence of mobile- versus fixed-bearing (Luring et al. 2006, Schuster et al. 2011, Matsumoto et al. 2017) and constraint where implant conformity may affect laxity (Ishii et al. 2005, Matsumoto et al. 2014, Yoshihara et al. 2016, Song et al. 2017, Wautier and Thienpont 2017). Further haze might occur due to differences in surgical technique, where gap-balancing and measured resection represent 2 different approaches to implant positioning and soft-tissue balancing, which is shown to have impact on the laxity (Lüring et al. 2009, Pang et al. 2011, Matsumoto et al. 2014, Clement et al. 2017).

We found no studies investigating to what extent preoperative anatomical conditions are reflected in the postoperative measurements of laxity. However, the preoperative mechanical axis has been reported not to correlate with intraoperative measurement of laxity at the end of surgery (Aunan et al. 2015). Preoperative and intraoperative factors not accounted for, such as mechanical alignment, severity of osteoarthritis, component alignment, and component rotation, might also introduce bias.

Tsukiyama et al. (2017) challenge the surgical gold standard of rectangular joint gaps in flexion and extension, as it was found that only medial coronal laxity, in opposition to lateral coronal laxity, in flexion influences outcome. This finding is in line with recommendations from some authors (Bellemans et al. 2006, Aunan et al. 2015, Risitano and Indelli 2017).

The range of soft-tissue laxity that favors both pain relief and optimal knee function following total knee arthroplasty (TKA) still needs clarification. Future studies using validated instruments should address the methodological issues of the reviewed studies, and might benefit from including performance-based outcome measurements. The combined impact of mechanical alignment and laxity on outcome should be investigated. However, this systematic review confirm that surgeons should measure and adjust ligament laxity intraoperatively in order to improve outcome following TKA.

## Supplementary Material

Supplemental Material

## Supplementary data

Table 1 and full search history is available as supplementary data in the online version of this article, http://dx.doi.org/10.1080/17453674.2018.1554400

AK: Primary draft of study protocol, database search and identification of full-text articles for assessment and primary draft of work. AO, PTN, MBL, AK: Revision and approval of study protocol, assessment of full-text articles, analysis and interpretation of data, revision and approval of work.

Professional and kind help from the university librarians Jette Frost Jepsen and Pernille Skou Gaardsted was highly appreciated.

*Acta* thanks Eirik Aunan and Kirill Gromov for help with peer review of this study.
