# Supplementary material for: Relationship between outcome scores and knee laxity following total knee arthroplasty: a systematic review
Source: Acta Orthop. 2018 Dec 20;90(1):46–52. doi: 10.1080/17453674.2018.1554400 (PMC6367957; doi:10.1080/17453674.2018.1554400)
Supplement: Supplemental Material [file IORT_A_1554400_SM1291.pdf]

Supplementary data

Table 1. Quality assessment of the included studies using MINORS

Authors and year	A	B	C	D	E	F	G	H	Total	Comments
Matsumoto et al. 2017	2	1	1	2	1	2	2	0	11	
Tsukiyama et al. 2017	2	1	1	2	1	2	2	0	11	
Graff et al. 2016	2	1	1	2	0	2	2	1	11	Underpowered (n = 24)
Nakahara et al. 2015	2	1	1	2	0	2	2	0	10	
Oh et al. 2015	2	1	2	2	1	2	1	0	12	Pre-surgery inclusion
Seah et al. 2012	2	2	2	2	2	2	1	0	13	Pre-surgery inclusion
Schuster et al. 2011	2	0	2	2	1	1	1	0	9	Pre-surgery inclusion
Seon et al. 2010	2	1	1	2	0	2	2	1	11	
Seon et al. 2007	1	2	2	2	2	2	1	0	12	Pre-surgery inclusion
Van Hal et al. 2007	2	1	1	2	1	2	2	0	11	
Jones et al. 2006	2	1	1	2	1	2	2	0	11	
Ishii et al. 2005	1	1	1	2	1	2	2	0	10	
Kuster et al. 2004	2	0	1	2	1	2	2	0	10	
Yamakado et al. 2003	1	0	1	2	1	2	2	0	9	Underpowered (n = 21)

The non-comparative part of the MINORS criteria was used (i.e., first 8 questions) as no studies analyzed the research question of this paper with use of a control group. The criteria of MINORS with 0 points when not reported, 1 when reported but not adequate, and 2 when reported and adequate. Maximum score is 16.

A. A clearly stated aim: the question addressed should be precise and relevant in light of the available literature.

B. Inclusion of consecutive patients: all patients potentially fit for inclusion (satisfying the criteria for inclusion) have been included in the study during the study period (no exclusion or details concerning the reasons for exclusion).

C. Prospective collection of data: data were collected according to a protocol established before the beginning of the study.

D. End-points appropriate to the aim of the study: unambiguous explanation of the criteria used to evaluate the main outcome, which should be in accordance with the question addressed by the study. In addition, the end-points should be assessed on an intention-to-treat basis.

E. Unbiased assessment of the study end-point: blind evaluation of objective end-points and double-blind evaluation of subjective end-points. Otherwise the reasons for not blinding should be stated.

F. Follow-up period appropriate to the aim of the study: the follow-up should be sufficiently long to allow the assessment of the main endpoint and possible adverse events.

G. Loss to follow-up less than 5%: all patients should be included in the follow-up. Otherwise, the proportion lost to follow-up should not exceed the proportion experiencing the major end-point.

H. Prospective calculation of the study size: information on the size of detectable difference of interest with a calculation of 95% CI, according to the expected incidence of the outcome event, and information on the level for statistical significance and estimates of power when comparing the outcomes.

 Acta Orthopaedica 2018; 89 (DOI: 10.1080/17453674.2018.1554400)

Search history

PubMed

Search	Query
#1	Search ((oks[tw]) OR KSS[tw]) OR HSS[tw]) OR hospital for special surgery score*[tw]) OR TKFQ[tw]) OR total knee function questionnaire*[tw]) OR JKOM[tw]) OR japanese knee osteoarthritis measurement*[tw]) OR university of california los angeles activity-level rating[tw]) OR (Knee injury and Osteoarthritis Outcome Score*[tw])) OR Oxford knee score*[tw]) OR koos[tw]) OR self-report*[tw]) OR "Self Report"[Mesh]) OR Patient Outcome Assessment*[tw]) OR patient reported outcome*[tw]) OR "Health Care Surveys"[Mesh]) OR "Patient Outcome Assessment"[Mesh]) OR PROM[tw]) OR PROMs[tw]) OR oxford score*[tw]) OR WOMAC*[tw]) OR McMaster Universities Osteoarthritis Index*[tw]) OR short form[tw]) OR shortform[tw]) OR SF-36[tw]) OR SF-12[tw]) OR SF-8[tw]) OR Forgotten Joint Score*[tw]) OR fjs[tw]) OR OKS-APQ[tw]) OR VR-12[tw]) OR Rand 12[tw]) OR EQ-5D[tw]) OR Euroqol 5[tw]) OR knee society score*[tw]) OR University of California Los Angeles Activity-level Rating*[tw]) OR UCLA[tw]) OR NRS[tw]) OR VAS[tw]) OR Numerical Rating scale*[tw]) OR Visual Analog Scale*[tw]) OR "Visual Analog Scale"[Mesh])) AND (((((((((("Postural Balance"[Mesh]) OR Balanc*[tw]) OR stabilit*[tw]) OR instabilit*[tw]) OR "Joint Instability"[Mesh]) OR Laxit*[tw]) OR "Range of Motion, Articular"[Mesh]) OR Range of Motion*[tw]) OR Flexibilit*[tw])) AND (((((Knee Replacement*[tw]) OR Knee arthroplast*[tw]) OR "Arthroplasty, Replacement, Knee"[Mesh]) OR knee reconst*[tw]) OR knee joint replacement*[tw])) Filters: Publication date from 2017/06/01 to 2018/12/31

Embase.com

No.	Query
#67	#6 AND #16 AND #66 AND [1-6-2017]/sd NOT [31-12-2018]/sd
#66	#17 OR #18 OR #19 OR #20 OR #21 OR #22 OR #23 OR #24 OR #25 OR #26 OR #27 OR #28 OR #29 OR #30 OR #31 OR #32 OR #33 OR #34OR #35 OR #36 OR #37 OR #38 OR #39 OR #40 OR #41 OR #42 OR #43 OR #44 OR #45 OR #46 OR #47 OR #48 OR #49 OR #50 OR #51 OR #52 OR #53 OR #54 OR #55 OR #56 OR #57 OR #58 OR #59 OR #60 OR #61 OR #62 OR #63 OR #64 OR #65
#65	'hospital for special surgery score'/exp
#64	'hospital for special surgery scor*'
#63	kss
#62	hss
#61	tkfq
#60	'total knee function questionnaire*'
#59	'japanese knee osteoarthritis measurement*'
#58	jkom
#57	'visual analog scale'/exp
#56	'numeric rating scale'/exp
#55	'knee society score'/exp
#54	'short form 36'/exp
#53	'patient-reported outcome'/exp
#52	'self report'/exp
#51	'oxford knee score'/exp
#50	'numerical rating scale*'
#49	'visual analog* scale*'
#48	vas
#47	nrs
#46	'university of california los angeles activity-level rating*'
#45	ucla:ti,ab
#44	'knee society scor*'
#43	'euroqol 5'
#42	'eq-5d'
#41	'rand 12'
#40	'vr 12'
#39	'oks apq'
#38	fjs
#37	'forgotten joint scor*'
#36	'sf-8'
#35	'sf-12'
#34	'sf-36'
#33	shortform
#32	'short form'
#31	'mcmaster universities osteoarthritis index*'
#30	'western ontario and mcmaster universities osteoarthritis index'/exp
#29	womac*
#28	'oxford scor*'
#27	proms
#26	prom
#25	'patient reported outcome*'
#24	'patient outcome assessment*'
#23	'self-report*'
#22	koos
#21	'oxford knee score'/exp
#20	'oxford knee scor*'
#19	'knee injury and osteoarthritis outcome scor*'
#18	'knee injury and osteoarthritis outcome score'/exp
#17	oks
#16	#7 OR #8 OR #9 OR #10 OR #11 OR #12 OR #13 OR #14 OR #15
#15	'range of motion*'
#14	'range of motion'/exp
#13	'knee function'/exp
#12	'joint laxity'/exp
#11	'knee instability'/exp
#10	laxit*
#9	instabilit*
#8	stabilit*
#7	balanc*
#6	#1 OR #2 OR #3 OR #4 OR #5
#5	'knee joint replacement*'
#4	'knee reconst*'
#3	'knee replacement*'
#2	'knee arthroplast*'
#1	'knee arthroplasty'/exp

Cochrane

ID	Search
#1	MeSH descriptor: [Arthroplasty, Replacement, Knee] explode all trees
#2	“knee reconst*”:ti,ab,kw (Word variations have been searched)
#3	“knee joint replacement*”:ti,ab,kw (Word variations have been searched)
#4	“Knee arthroplast*”:ti,ab,kw (Word variations have been searched)
#5	“Knee Replacement*”:ti,ab,kw (Word variations have been searched)
#6	#1 or #2 or #3 or #4 or #5
#7	Flexibilit*:ti,ab,kw (Word variations have been searched)
#8	MeSH descriptor: [Range of Motion, Articular] explode all trees
#9	“Range of Motion*”:ti,ab,kw (Word variations have been searched)
#10	Laxit*:ti,ab,kw (Word variations have been searched)
#11	MeSH descriptor: [Joint Instability] explode all trees
#12	instabilit*:ti,ab,kw (Word variations have been searched)
#13	stabilit*:ti,ab,kw (Word variations have been searched)
#14	Balanc*:ti,ab,kw (Word variations have been searched)
#15	MeSH descriptor: [Postural Balance] explode all trees
#16	#7 or #8 or #9 or #10 or #11 or #12 or #13 or #14 or #15
#17	#6 and #16
#18	MeSH descriptor: [Visual Analog Scale] explode all trees
#19	MeSH descriptor: [Health Care Surveys] explode all trees
#20	MeSH descriptor: [Patient Outcome Assessment] explode all trees
#21	MeSH descriptor: [Self Report] explode all trees
#22	oks:ti,ab,kw (Word variations have been searched)
#23	KSS:ti,ab,kw (Word variations have been searched)
#24	HSS:ti,ab,kw (Word variations have been searched)
#25	“hospital for special surgery score*”:ti,ab,kw (Word variations have been searched)
#26	TKFQ:ti,ab,kw (Word variations have been searched)
#27	“total knee function questionnaire*”:ti,ab,kw (Word variations have been searched)
#28	JKOM:ti,ab,kw (Word variations have been searched)
#29	“japanese knee osteoarthritis measurement*”:ti,ab,kw (Word variations have been searched)
#30	“university of california los angeles activity-level rating”:ti,ab,kw (Word variations have been searched)
#31	“Knee injury and Osteoarthritis Outcome Score*”:ti,ab,kw (Word variations have been searched)
#32	“Oxford knee score*”:ti,ab,kw (Word variations have been searched)
#33	koos:ti,ab,kw (Word variations have been searched)
#34	self-report*:ti,ab,kw (Word variations have been searched)
#35	“Patient Outcome Assessment*”:ti,ab,kw (Word variations have been searched)
#36	“patient reported outcome*”:ti,ab,kw (Word variations have been searched)
#37	PROM:ti,ab,kw (Word variations have been searched)
#38	PROMs:ti,ab,kw (Word variations have been searched)
#39	“oxford score*”:ti,ab,kw (Word variations have been searched)
#40	WOMAC*:ti,ab,kw (Word variations have been searched)
#41	“McMaster Universities Osteoarthritis Index*”:ti,ab,kw (Word variations have been searched)
#42	“short form”:ti,ab,kw (Word variations have been searched)
#43	shortform:ti,ab,kw (Word variations have been searched)
#44	SF-36:ti,ab,kw (Word variations have been searched)
#45	SF-12:ti,ab,kw (Word variations have been searched)
#46	SF-8:ti,ab,kw (Word variations have been searched)
#47	“Forgotten Joint Score*”:ti,ab,kw (Word variations have been searched)
#48	fjs:ti,ab,kw (Word variations have been searched)
#49	OKS-APQ:ti,ab,kw (Word variations have been searched)
#50	VR-12:ti,ab,kw (Word variations have been searched)
#51	“Rand 12”:ti,ab,kw (Word variations have been searched)
#52	EQ-5D:ti,ab,kw (Word variations have been searched)
#53	“Euroqol 5”:ti,ab,kw (Word variations have been searched)
#54	“knee society score*”:ti,ab,kw (Word variations have been searched)
#55	“University of California Los Angeles Activity-level Rating*”:ti,ab,kw (Word variations have been searched)
#56	UCLA:ti,ab,kw (Word variations have been searched)
#57	NRS:ti,ab,kw (Word variations have been searched)
#58	VAS:ti,ab,kw (Word variations have been searched)
#59	“Numerical Rating scale*”:ti,ab,kw (Word variations have been searched)
#60	“Visual Analog Scale*”:ti,ab,kw (Word variations have been searched)
#61	#18 or #19 or #20 or #21 or #22 or #23 or #24 or #25 or #26 or #27 or #28 or #29 or #30 or #31 or #32 or #33 or #34 or #35 or #36 or #37 or #38 or #39 or #40 or #41 or #42 or #43 or #44 or #45 or #46 or #47 or #48 or #49 or #50 or #51 or #52 or #53 or #54 or #55 or #56 or #57 or #58 or #59 or #60
#62	#17 and #61 Publication Year from 2017 to 2018
